# The Immune Microenvironment of Breast Cancer Progression

**DOI:** 10.3390/cancers11091375

**Published:** 2019-09-16

**Authors:** Helen Tower, Meagan Ruppert, Kara Britt

**Affiliations:** 1 Breast Cancer Risk and Prevention, Peter MacCallum Cancer Centre, 305 Grattan St, Melbourne, Victoria, VIC 3000, Australia; helen.m.tower@gmail.com (H.T.); Meagan.Ruppert@petermac.org (M.R.); 2 The Sir Peter MacCallum Department of Oncology, University of Melbourne, Parkville, Victoria, VIC 3000, Australia

**Keywords:** breast cancer, immune microenvironment, DCIS, ADH

## Abstract

Inflammation is now recognized as a hallmark of cancer. Genetic changes in the cancer cell are accepted as the match that lights the fire, whilst inflammation is seen as the fuel that feeds the fire. Once inside the tumour, the immune cells secrete cytokines that kick-start angiogenesis to ferry in much-needed oxygen and nutrients that encourage the growth of tumours. There is now irrefutable data demonstrating that the immune contexture of breast tumours can influence growth and metastasis. A higher immune cell count in invasive breast cancer predicts prognosis and response to chemotherapy. We are beginning now to define the specific innate and adaptive immune cells present in breast cancer and their role not just in the progression of invasive disease, but also in the development of pre-invasive lesions and their transition to malignant tumours. This review article focusses on the immune cells present in early stage breast cancer and their relationship with the immunoediting process involved in tumour advancement.

## 1. Introduction 

The immune system protects the host from pathogens and toxic, allergenic or foreign substances. It is broadly classified into two lines of defence: innate immunity and adaptive immunity. Innate immunity is comprised of the initial immune response, occurring within hours of encountering a foreign antigen, and is antigen-independent (non-specific). On the other hand, adaptive immunity is antigen-dependent and pathogen-specific, but requires approximately 4-7 days to mount a full active response. It is well accepted that the immune system has an integral role in shaping the evolution of cancer through the process of immunoediting. Testament to this, immunotherapy now forms part of some cancer treatments, rallying the body’s immune system to fight cancer. Checkpoint inhibitors, for example, have been developed to target and block the immune checkpoint proteins CTLA-4, PD-1 and PD-L1, which are upregulated on tumour cells and immune cells and restrict the immune system from attacking the tumour. The checkpoint inhibitor therapies reactivate the T cells, leading to durable responses and long-term survival in lung cancer and melanoma. Here we discuss the role of the immune system in breast cancer (BCa), including invasive cancers and the pre-invasive in-situ lesions, where recent work shows the innate and adaptive cells are already activated. 

## 2. Lesions of the Breast

**Figure 1:**
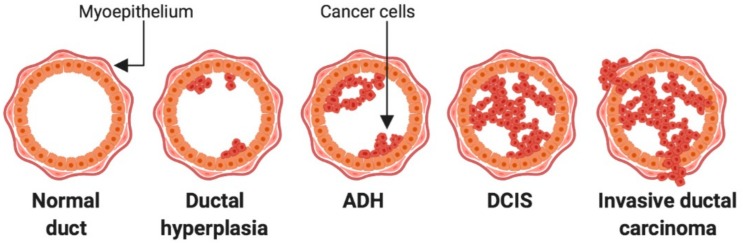
Stages of breast cancer development. Tumour cell initiation and expansion within the mammary ducts characterises atypical ductal hyperplasia (ADH). This progresses to ductal carcinoma in situ (DCIS), which is identified as a complete filling of the mammary duct with tumour cells. Once the myoepithelium is breached and tumour cells escape beyond the mammary duct confinement, the cancer is classified as an invasive ductal carcinoma [7].

Carcinoma of the breast can arise from either the lobular or the ductal epithelium. Lobular carcinomas are less prevalent than ductal, accounting for 4–10% of diagnoses from breast biopsies [1]. Before BCa reaches the invasive stage at which point it can spread to the rest of the body, it is referred to as a pre-invasive lesion (Figure 1). In pre-invasive lesions, the cancerous cells are confined to the ducts or lobules from which they originate and have not yet broken the basement membrane [2]. The pre-invasive lesions in ductal carcinoma are categorized as either atypical ductal hyperplasia (ADH) or ductal carcinoma in situ (DCIS). It can be difficult to histologically distinguish ADH lesions from that of low-grade DCIS, as the lesions look similar and ADH is mostly identified through failing to meeting the diagnostic criteria for DCIS [3,4]. Ducts exhibiting abnormal proliferation that receive a diagnosis of ADH are partially or completely filled with uniform and polarized cells. These cells are hyperchromatic, and the extent of proliferation is greater than that found in usual ductal hyperplasia [4]. Ducts affected by ADH are small, typically defined as less than or equal to 2 millimetres (mm) in size, and are usually found alone or in small, clustered foci [3]. Not all ADH lesions will progress to carcinoma [5], however women with a diagnosis of ADH are four times more likely to develop BCa [6]. 

DCIS lesions are characterized into low, intermediate, and high grade. The grades are distinguished by cellular features including the presence of calcifications and necrosis within the duct, the regularity and uniformity of the cells and their nuclei, and the extent of proliferation causing distortion of the duct [8]. Irregularity of the tumour cell nuclei, mitotic figures, and the extent of necrosis within the duct all dictate higher lesion grading [9]. DCIS lesions are typically surgically removed, but their diagnosis confers a risk of both DCIS recurrence and progression to invasive disease. This occurs for each DCIS grade, however the risk of recurrence or progression is highest in the high-grade lesions, and lowest in the low-grade lesions [10,11,12,13]. 

Tumours are characterized as invasive ductal carcinoma (IDC) once the cells are no longer confined to the affected duct, but have broken through the basement membrane and subsequently have invaded the surrounding stroma [2]. The presence of hormone or growth factor receptors can divide the invasive cancers into distinct subtypes. These include estrogen receptor-positive (ER+), human epidermal growth factor receptor 2 (HER2)-positive, and triple negative (TNBC) BCa [14]. TNBC expresses neither hormone nor growth factor receptors. BCa can also be classified by molecular characteristics into luminal (which can be ER+ or ER− and also ER+HER2+), or HER2+ (which expresses amplification of the human epidermal growth factor receptor 2 (HER2) gene, but are negative for ER). Finally, there are 2 subtypes that lacks all growth factors and are referred to as Basal and Claudin low [15]. 

## 3. The role of the Immune System in Cancer 

Alongside the traditional hallmarks of cancer such as unregulated cell growth and apoptosis evasion, immune-manipulating mechanisms are also considered pivotal characteristics of cancer cells [16]. Tumours have the ability to influence their immune microenvironment either by exerting immunosuppressive signalling, evading immune recognition, or fuelling tumour-promoting inflammation as a means of driving cancer progression. Given the appropriate conditions, leukocyte activation initiated by mutated cells can advance neoplastic transformations into malignant tumour cells [16]. 

**Figure 2:**
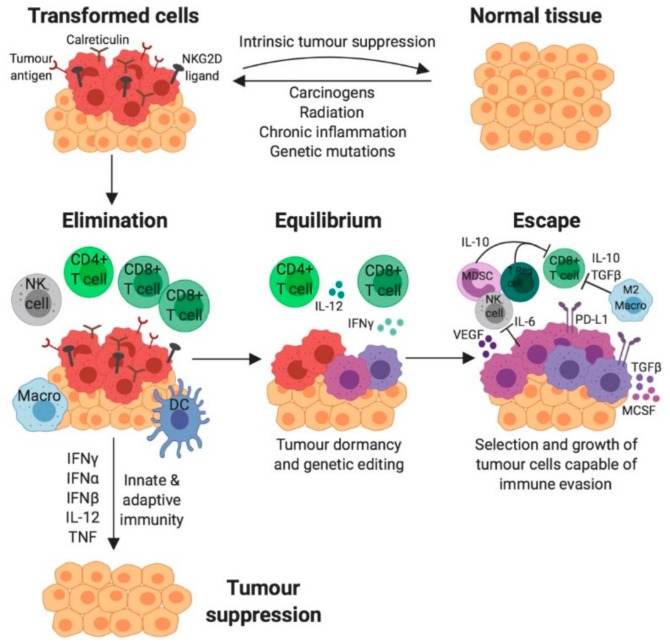
The three phases of cancer immunoediting. Normal cells transition to tumour cells expressing specific tumour antigens, calreticulin, and NKG2D ligands if subject to oncogenic mutational transformation. Elimination is the first phase of cancer immunoediting, where the cells of the innate and adaptive immune system are recruited to the site of the tumour antigens and attempt to destroy tumour cells via immune attack mechanisms (including secretion of cytokines IFNγ, IFNα, IFNβ, IL-12 and TNF). Any persisting tumour cells enter the second phase, equilibrium, where selection pressures instigate new tumour cell genetic variants. These genetic modifications allow tumour evasion of the immune system and promotion to the third phase, escape, where tumour cells progressively develop and become clinically detectable as a palpable mass. Immune evasion is influenced by factors including tumour cell PD-L1 upregulation, secretion of immuno-inhibitory cytokines (IL-6, IL-10, TGFβ and MCSF) and recruitment of inhibitory immune cells (M2 macrophages, Regulatory T (TReg) cells and Myeloid-derived suppressor cells (MDSCs)) that abrogate immune-mediated tumour cell killing via inhibition of Natural Killer (NK) cells and CD8+ T cells. Tumour cells also experience a downregulation of tumour antigen, calreticulin and NKG2D ligands, so are less susceptible to immune recognition [19].

This gives rise to the cancer immunoediting hypothesis. Here, it is postulated that the immune system exerts both host-protective and tumour-stimulating actions [17]. Cancer immunoediting is multifaceted and composed of three fundamental phases: elimination, equilibrium and escape (Figure 2) [18]. Initially, tumour-specific antigens are recognised by the innate and adaptive arms of the immune system and elicit a pro-inflammatory response [18]. The cancer immunosurveillance network acts in cohesion to eliminate developing tumour cells, thereby preventing further tumourigenesis. Tumours only progress to the equilibrium phase if immunosurveillance is unsuccessful or impaired. Subsequently, cancerous cells persisting habitually in equilibrium with their microenvironment are more equipped to mutate and produce new populations of tumour variants [18]. Modifications to tumour cells can allow them to ultimately employ immunosuppressive mechanisms, thus evading and essentially escaping the immune system in the final phase [18]. These immunologically sculpted tumours grow with fewer selective pressures, actively induce an immunosuppressive microenvironment and become evident clinically.

## 4. The Innate and Adaptive Immune System

The immune system is an organism’s natural defence mechanism that provides protection from a plethora of pathogens, infections and diseases. Immune regulation is tightly controlled, which enables appropriate recognition and response to foreign threats whilst avoiding unwanted inflammation towards healthy tissue and the body’s natural microbial flora. The immune system is composed of a dynamic network of cells, tissues and organs that broadly function in two lines of defence: innate and adaptive immunity [20]. This division of the immune system is tailored directly towards the pathogenic threat encountered, and also confers immunological memory where long-lasting protection against the specific pathogen is established [20]. Both arms of the immune system are emerging to play key roles on BCa development and progression.

## 5. Tumour Infiltrating Lymphocytes (TILs) and BCa

Tumour infiltrating lymphocytes (TILs) are immune cells that have migrated to the tumour tissue and the local microenvironment. This population is indicative of an immune response generated by the patient against the malignancy. In TNBC and HER2+ disease in particular, the presence of TILs has been shown to correlate with a good prognosis and good response to chemotherapy. The relationship has not been as definitely proven for ER+ disease, indicating that the luminal subtypes may be less immunogenic than the others. This indicates that simple TIL counts are not as effective as a prognostic marker in these tumours [21,22,23]. TILs have also been found to be a prognostic indicator for higher rates of pathological complete responses (pCRs) to neoadjuvant chemotherapy [24,25,26,27]. 

Whilst TILs can be present within or around the tumour, TIL assessment is primarily concerned with stromal TILs counted in H&E-stained tumour sections as stated by The International TILs Working Group. While TILs in each compartment together constitute the population of lymphocytic infiltration and may contribute to prognostic significance, the majority of TILs are found in the stroma. Intratumoural TILs are difficult to quantify and low concordance between different scorers of the same sample have been reported [28]. To quantify the number of stromal TILs, the guidelines state that one should count the proportion of TILs in the stromal compartment in the visual field. Experts in the field have developed guidelines and tutorials for assessing TILs in invasive cancers and metastases, as well as DCIS lesions. 

## 6. The Immune Regulation in Invasive BCa 

TILs have been found to be elevated in primary invasive cancers compared to metastases. The TIL populations across BCa in general are predominantly made up of T lymphocytes, and in particular CD8+ cytotoxic T lymphocytes (CTLs). Due to this fact, CD8+ cells are a robust immune prognostic marker for the outcome of BCa patients, particularly the TN and HER2+ subtypes, because they represent an active, adaptive immune response to the neoantigens on the surface of the tumour cells and correlate positively with improved survival [29]. 

CTLs have the capacity to differentiate further into tissue-resident memory T (T_RM_) cells that exist within the breast tissue without recirculating systemically. T_RM_ cells express high levels of immune checkpoint molecules that contribute to tumour elimination and have been shown to be actively involved in BCa immunosurveillance. T_RM_ status has been shown to be an even greater prognostic marker than CD8+ cells alone, and is significantly associated with improved TNBC patient survival [30]. The T helper cells present during acute inflammation are predominantly T helper cell type 1 (Th1) polarized and secrete cytokines such as IFNγ, TNFα and IL-2 which act to limit tumour growth, promote antigen processing and presentation, and activate macrophages. The T helper cells present during chronic inflammation and cancer are type 2 (Th2) polarized and express IL-4, IL-5, IL-6, IL-10 and IL-13 which inhibit T cell-mediated cytotoxicity [31]. T regulatory lymphocytes (Tregs) are characterised as T lymphocytes which are both CD4+ and FOXP3+ and have immunosuppressive functions. Tregs normally help to protect against autoimmunity [32]. In the context of breast carcinomas, these immune cells are largely agreed to contribute to the pro-tumour immune response and assist the tumour in subsequent immune escape, so are thus associated with a poor prognosis [21,33]. These lymphocytes allow the progression of the tumour by expressing inhibitory factors that inhibit the anti-tumour Th1 response [33]. 

**Figure 3:**
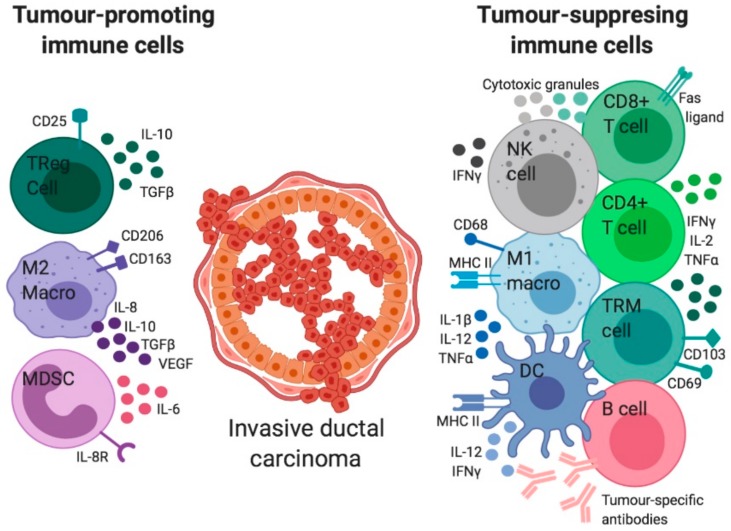
The immune microenvironment of invasive ductal carcinoma. Subsets of the immune system can elicit both tumour-promoting and tumour-suppressing effects. Immune inhibition of tumours is largely driven by the activity of CD8+ T cells, CD4+ T cells, Tissue-resident T (T_RM_) cells, B cells, Natural Killer (NK) cells, M1 macrophages and dendritic cells (DC). The tumour-fighting immune landscape produces cytokines that inhibit tumour development (including IFNγ, TNFα, IL-1β, IL-2 and IL-12). CD8+ T cells and NK cells also secrete cytotoxic granules that trigger tumour cell apoptosis. Similarly, B cells secrete tumour-specific antibodies that target tumour cells for elimination. In contrast, the immune stimulation of tumours is promoted by Regulatory T (TReg) cells, M2 macrophages and Myeloid-derived suppressor cells (MDSCs), which act to suppress their anti-tumour immune counterparts and facilitate tumour growth. These cells release immuno-inhibitory pro-tumour cytokines (TGFβ, VEGF, IL-6, IL-8 and IL-10) [37,38,39,40,41].

In addition to T cells there are many other immune cell types that infiltrate breast cancers including macrophages, NK cells, and dendritic cells (DCs) (Figure 3) [19,34,35]. In brief, CD4+ T helper, CD8+ CTLs, NK cells, M1 macrophages, and DCs are protective against tumour growth [36]. Conversely, CD4+ FOXP3+ Th2 cells, M2 macrophages, and myeloid-derived suppressor cells (MDSCs) can drive tumour growth [36]. 

Tissue-resident macrophages are typically found along the ductal system in the stroma of the normal breast, and are present prior to the development of any malignancy [42,43]. These are some of the first immune cells to encounter tumour cells when they begin to form a hyperplastic or neoplastic growth. The macrophages associated with tumours are referred to as tumour associated macrophages (TAM) and their infiltration accompanies a worse prognosis in many cancers [44,45]. TAMs in invasive BCa have been shown to express higher levels of the transcription factor hypoxia-inducible factor 2α (HIF-2α) in comparison to macrophages from the normal breast [46]. HIF-2α along with HIF-1α from the tumour cells [47] activate the expression of vascular endothelial growth factor (VEGF) which stimulates angiogenesis [48,49]. By vascularizing an early tumour, the TAMs ensure that the tumour receives the nourishment it requires for malignant growth and metastasis. In support of this positive feedback, microvessel density and VEGF expression have been found to be significantly correlated with TAM density in IDC [50]. 

Cytokines from the tumour microenvironment are also key players in this process, as they can induce phenotypic changes in macrophages. IL-10 and TGF-β switch the macrophages from an M1-like (proinflammatory or classically activated) state to an M2-like (anti-inflammatory or alternatively activated) state. M1 macrophages elicit anti-tumour immune signaling and are associated with tumour killing capacity. Conversely, M2 macrophages exert pro-tumour effects and are associated with fibrosis and the production of matrix proteins [51] as well as angiogenesis, metastasis, and the suppression of adaptive immunity [52,53]. Human BCa cell lines cultured in vitro are able to polarise macrophages towards the M2 phenotype [54]. In BCa patient samples, M2 macrophages in the stroma correlate with the presence of a lesion [54]. Retrospective studies of preserved human tumours have demonstrated that M2 macrophages are significantly associated with poor prognosis in both ER- and ER+ tumours [21]. 

MDSCs are a collection of progenitor and immature myeloid-lineage cell types which serve as a brake on immune system activation [55]. High levels of MDSCs have been identified as a poor prognostic marker for many cancers, and most likely participate in the pro-tumourigenic pathway through the suppression and inhibition of the host anti-tumour immune response [55,56]. BCa patients have higher circulating MDSC counts than their normal-matched counterparts [57]. Greater quantities of MDSCs isolated from the blood of these patients correlated with poor prognosis, and when cultured with T cells in vitro they were able to significantly inhibit proliferation of the lymphocytic population in comparison to MDSCs derived from normal subjects [57]. Looking at early-stage breast cancer, patients with greater neutrophil (a type of MDSC) counts had a higher neutrophil to lymphocyte ratio and were more likely to relapse [58].

DCs express MHC Class II and can present their antigenic peptides to CD4+ T cells. They prime tumour specific effector T cells to attack the tumour and are thought to play an important role in shaping the host response to the cancerous cells. DC maturation and survival are impaired in invasive tumours and the infiltration of plasmacytoid DCs (pDCs) in primary BCa is correlated with poor clinical outcome. This indicates that pDCs contribute to BCa progression [59].

Natural killer (NK) cells are unique in that they have both innate and adaptive immune properties [60]. NK cells participate in the anti-tumour immune response through the production of pro-inflammatory cytokines, which recruit and induce proliferation of other immune cells [61]. NK cells can also directly mediate anti-tumour immunity by killing the tumour cells themselves without prior sensitization, so therefore play an active role in cancer immunosurveillance [62,63]. However, the ability of NK cells to recognize and kill tumour cells is impaired in cancer patients, as tumour NK cells exhibit an inhibitory phenotype characterised by the expression of inhibitory markers [64,65,66]. In BCa patients, NK cell dysfunction correlates with tumour progression and invasiveness [65,67]. 

B lymphocytes are CD20+ adaptive immune cells which confer humoral immunity through the production and secretion of antibodies, which are made of the protein immunoglobulin and recognise specific tumour-antigens. Antibodies bind to these antigens and can inhibit the functionality of the receptor or ligand they are bound to. Additionally, antibodies can signal to other cancer-killing cells that they are bound to a tumour cell, thereby activating them to eliminate tumour cell populations [68]. B cells participate in immunity alongside the T cell response through their ability to present antigen and co-stimulatory molecules to these lymphocytes [69]. Furthermore, B cells can be found in the breast milk secreted by the lactating normal breast [42,43]. In IDC, B lymphocytes and immunoglobulin gene expression signatures have been associated with a favourable prognosis in retrospective studies [70,71]. Though this supports an anti-tumour role for B cells and antibodies, other studies have associated them with poor prognostic factors in BCa. Human BCa cells can induce a regulatory phenotype in B cells, instigating production of transforming growth factor beta (TGF-β), a cytokine that stimulates CD4+ T cells to become immunosuppressive T regulatory cells [72].

Although not strictly an immune cell, fibroblasts are present within the stromal microenvironment of the breast and serve to produce the extraceullular matrix (ECM) proteins (in particular collagen). They can also manufacture and respond to cytokines, allowing them to cooperate with the immune cells within the stromal microenvironment. However, fibroblasts can control epithelial cell polarity, proliferation, and to some extent, tumourigenic potential. Cancer associated fibroblasts (CAFs) have been shown to drive increased tumour growth compared to normal fibroblasts [73]. They contribute to cancer cell survival and progression by secreting high levels of nutrient-rich ECM proteins, or ECM-degrading proteases. These can promote persistent chronic inflammation within the tumour microenvironment and inducing the epithelial mesenchymal transition (EMT) of tumour cells [74,75,76,77].

CAFs have the capacity to produce pro-inflammatory cytokines. These CAF-secreted cytokines disrupt the normal cytokine balance to stimulate tumour growth by initiating angiogenesis and inhibiting CTLs. CAFs have been shown to secrete high levels of the pro-inflammatory cytokines IL-1β, IL-8, IL-10, tumour necrosis factor-alpha (TNFα), monocyte chemoattractant protein-1 (CCL2), stromal derived factor-1 (CXCL12) and interferon-beta (IFNβ) [78,79]. The importance of the CAFs in cancer growth has been highlighted by genetic analyses showing that their gene expression profiles are very different to normal breast fibroblasts. Moreover, the expression profiles of CAFs taken from tumours with poor (increased recurrence and shorter disease-free survival) versus good (reduced recurrence and longer disease-free survival) outcome are also very different [80]. The good-outcome fibroblasts were enriched for immune modulators such as those involved in the Th1 immune response. This includes expression of T cell receptor complexes (CD8a, CD247, CD3D), MHC class I protein binding and granzyme A/B activity. The poor-outcome stroma had increased levels of hypoxia and angiogenesis and decreased chemokines that stimulate NK migration and T cell survival [80].

## 7. Immune Regulation of DCIS

The pre-invasive DCIS stage of BCa also exhibits significant immune infiltration. Gorringe and colleagues showed that TILs are present in high grade DCIS lesions, with smaller numbers also observed in low and intermediate grade lesions [81]. A global study of 53 mastectomy samples demonstrated that T cell, B cell and macrophage levels were all elevated in DCIS compared with the normal breast and remained elevated across subsequent cancer progression [82]. Similarly, neutrophils have been found to be significantly higher in the breast of women with DCIS than in the normal breast [83]. Clinical research assessing specific subsets of immune cells in DCIS has indicated that CD68+ macrophages (in particular the M2 macrophages), CD4+ T cells and CD20+ B cells were elevated in the high-grade DCIS cases compared to low [84]. 

CAFs are also thought to play a role in the transition of DCIS to IDC via their secretion of factors which modify the surrounding stromal matrix. When fibroblasts sourced from the normal breast or IDC were injected alongside a DCIS cell line in xenografts, the IDC-derived fibroblasts (or CAFs) elicted a significant increase in tumour weight whilst normal fibroblasts had no effect on xenograft progression [85]. One mechanism through which CAFs may be accomplishing this in early BCa development is through the production of IL-10, which not only stimulates M2 polarisation of TAMs but additionally serves to modulate T cell and NK cell phenotypes [54,86]. Osula et al. have demonstrated that CAFs significantly upregulate expression of IL-6 in comparison to their normal counterparts. By culturing these fibroblasts with DCIS cell lines, Osula and colleagues revealed that DCIS cells grow faster, and that this growth is inhibited with treatment of an IL-6 neutralising antibody [87]. Paracrine IL-6 signalling between malignant cells and pro-tumour fibroblasts may thus be influential in the progression of DCIS to IDC.

Recurrent DCIS describes the reappearance of additional DCIS lesions after the primary diagnosis and treatment and/or progression to invasive disease [9]. The highest risk of DCIS recurrence correlates with patients displaying both low T cell numbers and elevated macrophages [84]. Of these recurrent cases, immunosuppressive CD206+ M2 macrophages were also prevalent, thus suggesting that their anti-inflammatory effects may abrogate tumour-fighting T cell functions in these early lesions [84]. Illustrating the pro-tumourigenic role of macrophages, mouse models transplanted with pre-invasive breast cancer cell lines have shown that metastatic progression is inhibited when the macrophages are depleted prior to transplantation [88]. When directly compared with invasive BCa, the inflammatory response to the malignant cells in DCIS is highly active. There are significantly more CTLs in DCIS expressing granzyme B and IFNγ, marking these cells as activated, effector cytotoxic T cells [83]. In the same study, the diversity of T cell receptor clonotypes were found to be significantly higher in DCIS than in IDC. 

During a chronic infection, CD8+ T cells undergo a hierarchical loss of function and increase the expression of coinhibitory receptors in a process called exhaustion. Importantly, targeting coinhibitory receptors such as PD-1 and CTLA-4 using monoclonal antibodies, alone or in combination, has proven to be effective in restoring the function of exhausted T cells. When DCIS and IDC were compared, the T cell immunoglobulin and ITIM domain (TIGIT) co-inhibitory receptor was found to have higher expression in T cells from DCIS patients compared to HER2+ and TNBC IDC. However, PD-L1 was almost undetectable in DCIS and increased in IDC. CTLA-4 was also higher in T cells from IDCs compared with DCIS [83]. Together this indicates that the immune microenvironment becomes suppressive during invasive progression. but that each of the checkpoint molecules may play a distinct role. 

## 8. Immune Regulation of Hyperplasia

The immune regulation of early hyperplastic breast tumourigenesis is understood to a considerably lesser degree than that of DCIS. Limited published data exists surrounding the immune infiltrate in ADH of the breast. Gorringe and colleagues have shown that in DCIS a higher Fraction of Genome Altered compared to normal (measured by copy number variation analysis) correlates with a higher infiltration of TILs in DCIS [81]. They have not yet assessed TIL numbers in ADH compared to the normal breast, but have shown that ADH lesions exhibit aneuploidy, loss of heterozygosity, gross chromosomal rearrangement (such as amplifications and large-scale deletion) and methylation changes. This indicates that the genomic changes present may activate the immune system early. In support of this, human studies in endometrial cancer have demonstrated that significant immune involvement enhanced the proliferative rate of hyperplastic tissue [89]. In particular, macrophage numbers and the inflammatory cytokines they produce were significantly associated with early malignancies and tumourigenesis. 

Whilst the immune composition of ADH has not yet been explored, increased CD4+ T cells, CTLs and B cells have been observed in lobules with lobulitis [90]. Similarly, normal breast tissue from women with high breast density (which confers a 4-6-fold increased risk of BCa) exhibits increased macrophages, DCs, B cells and CD4+ T cells. High-density tissue also disaplayed increased IL-6 and IL-4 secretion, thereby suggesting pro-tumour Th2 polarization [91]. The lack of information in hyperplastic lesions may be due their small size (≤2 mm) [92,93,94] and close relationship to low grade DCIS. This means the ADH samples are only collected for diagnostic purposes and not research. It is also notable that there is a lack of concordance amongst pathologists in differentiating low-grade DCIS and ADH [94]. Further analysis of the immune landscape of pre-invasive lesions including ADH will hopefully reveal whether the microenvironment assists at this earliest stage of tumour escape from immune regulation.

Fibroblasts may also play a role in the earliest stages of tumour growth. This notion has been suggested by work demonstrating that stromal-specific inactivation of TGFβ-RII leads to pre-invasive prostate cancer lesions in mice [95], and that stromal phosphatase and tensin homolog (PTEN) loss can drive BCa growth [96]. In addition to stimulating the growth of early hyperplastic cells, CAFs may participate in the loss of epithelial characteristics of ductal lesions. Fibroblasts isolated from ADH-affected breast tissue exhibited an activated phenotype mirroring that of CAFs isolated from DCIS. When cultured with the BCa cell line MCF-7, these ADH-associated fibroblasts induced decreased expression of the epithelial protein e-cadherin and increased expression of the mesenchymal protein vimentin [97]. This indicates that even when precursor cells are merely hyperplastic, activation of fibroblasts under appropriate conditions may prime the lesion towards malignant transformation.

## 9. Immune-Based Therapies for BCa Growth and Progression

As mentioned above, it is well-established that in both IDC and DCIS, high numbers of stromal lymphocytes serve as a good prognostic factor in TNBC and HER2+ disease. This opens the door to potentially utilize immunotherapies to mobilize the immune system against BCa. The PD-1/PD-L1 inhibitory pathway is one of the most intensively investigated avenues in the development of immune-based therapeutics. PD-L1 is known to be expressed by primary and metastatic IDCs and has been identified as a poor prognostic indicator in these patients [98,99,100], likely serving to downregulate the T cell response to tumour cells. As TNBC has limited successful therapies, anti-PD-L1 therapy represents a promising new treatment option. In vivo models of TNBC demonstrate that treatment with antibodies targeting PD-L1 expressed on tumour cells reduces tumour volume whilst increasing tumour immunogenicity [101]. PD-L1 inhibition has additionally been investigated for use in DCIS. Expression of the immunosuppressive ligand is far less common in this earlier stage, though when expression is observed, it is most commonly in HER2+ lesions [81,102]. Several trials are currently underway investigating the use of anti-PD-L1 therapies both alone and in combination with HER2-specific treatments, as reviewed by Ubago et al. [103].

Combination immunotherapies have also been proven as potentially efficacious novel TNBC treatments. TNBC often upregulates activity of MEK, which contributes to an overactivation of the Ras/MAPK pathway and thus serves as a poor prognostic indicator for recurrence and survival in these patients. However, T cells also use MEK signaling for proliferation, activation, and differentiation, so simply inhibiting MEK activity reduces the potential utility of recruiting the immune system. When MEK inhibition was combined with agonist antibodies to activating receptors on T cells, or with anti-PD-1/PD-L1 therapies, survival and tumour size significantly improved in xenograft TNBC models [101,104].

Immunotherapies tend to particularly target T cells and the adaptive immune response. Innate immunity represents an alternative route through which to pursue immune-based therapies. One such novel therapy is anti-CSF1R, which inhibits the receptor found on TAMs responsible for their recruitment and M2 activation as pro-tumourigenic immune cells. Mixed data exists concerning the role of CSF1R inhibition and tumour growth in mice, with some studies demonstrating significant reductions in tumourigenesis and others showing limited effectiveness or stimulated cancer metastasis [105,106]. This avenue of innate immune modulation in cancer therapies therefore remains somewhat elusive, and requires further research to shed light on any possible therapeutic benefits. 

## 10. Conclusions

The role of the immune microenvironment in BCa is becoming clearer. Infiltrating immune cells in invasive lesions are predominantly T lymphocytes, and in particular CTLs. The CD8+ CTLs are now viewed as a robust immune prognostic marker for the outcome of TN and HER2+ BCa patients. There are additional innate and adaptive cells that infiltrate BCa or remain in close proximity in the stromal microenvironment. CD4+ T helper, CD8+ CTLs, NK cells, M1 macrophages, and DCs are likely protecting against tumour growth whilst the CD4+ FOXP3+ Th2 cells, M2 macrophages, and myeloid-derived suppressor cells (MDSCs) simulate tumour growth. The cytokines present at the tumour site are also key players in this process, often controlling the infiltration as well as activation and polarization state of the immune cells. CAFs have altered gene expression profiles and function compared to normal breast fibroblasts and can drive increased tumour growth by aiding cancer cell survival and progression and by secreting high levels of nutrient-rich ECM proteins, or ECM-degrading proteases. Less is known about the pre-invasive stages of BCa development including DCIS and ADH. However, TILs are present in high-grade DCIS lesions, less so in low- and intermediate-grade lesions. Those pre-invasive lesions with the highest risk of recurrence correlates have been show to exhibit low T cell numbers and elevated macrophages. The TIL numbers at this time may be correlated with the level of genetic changes that are measured within the lesions. As ADH lesions already exhibit significant genetic changes (aneuploidy, loss of heterozygosity, gross chromosomal rearrangement and methylation changes) it is expected that the immune system will already be activated and in hyperplastic endometrial cancer this has been shown. As we increase our understanding of the earliest stages of BCa development and the interaction with the immune system, we will begin to define whether immune therapies can be delivered earlier for better disease control.
